# Identification and characterisation of temporal abundance of microRNAs in synovial fluid from an experimental equine model of osteoarthritis

**DOI:** 10.1111/evj.14456

**Published:** 2025-01-08

**Authors:** Marie Walters, Kerstin Skovgaard, Peter M. H. Heegaard, Yongxiang Fang, Yalda A. Kharaz, Louise Bundgaard, Lene T. Skovgaard, Henrik E. Jensen, Pia H. Andersen, Mandy J. Peffers, Stine Jacobsen

**Affiliations:** ^1^ Department of Veterinary Clinical Sciences University of Copenhagen Taastrup Denmark; ^2^ Department of Biotechnology and Biomedicine Technical University of Denmark Kgs. Lyngby Denmark; ^3^ Department of Health Technology Technical University of Denmark Kgs. Lyngby Denmark; ^4^ Centre for Genomic Research, Institute of Infection, Veterinary & Ecological Sciences University of Liverpool Liverpool UK; ^5^ Department of Musculoskeletal Ageing Science, Institute of Life Course and Medical Sciences University of Liverpool Liverpool UK; ^6^ Department of Public Health University of Copenhagen Copenhagen K Denmark; ^7^ Department of Veterinary and Animal Sciences University of Copenhagen Frederiksberg Denmark; ^8^ Department of Anatomy, Physiology and Biochemistry Swedish University of Agricultural Sciences Uppsala Sweden

**Keywords:** horse, microRNA, osteoarthritis, post‐transcriptional gene regulation, reverse transcription quantitative polymerase chain reaction, small RNA sequencing, synovial fluid

## Abstract

**Background:**

MicroRNAs, a class of small noncoding RNAs, serve as post‐transcriptional regulators of gene expression and are present in a stable and quantifiable form in biological fluids. MicroRNAs may influence intra‐articular responses and the course of disease, but very little is known about their temporal changes in osteoarthritis.

**Objectives:**

To identify miRNAs and characterise the temporal changes in their abundance in SF from horses with experimentally induced osteoarthritis. We hypothesised that the abundance of miRNA would change during disease progression.

**Study design:**

In vivo experiments.

**Methods:**

RNA extracted from synovial fluid obtained sequentially (Day 0, 28 and 70) from nine horses with experimentally induced osteoarthritis was subjected to small RNA sequencing using the Illumina Hiseq 4000 sequencing platform. Differentially abundant miRNAs underwent further validation and mapping of temporal abundance (Day 0, 14, 17, 21, 28, 35, 42, 49, 56, 63 and 70 days after osteoarthritis induction) by microfluidic reverse transcription quantitative real‐time PCR. Bioinformatic analyses were performed to predict potential biological associations and target genes of the differentially abundant microRNAs.

**Results:**

Small RNA sequencing revealed 61 differentially abundant microRNAs at an early osteoarthritis stage (Day 28), and subsequent reverse transcription quantitative real‐time PCR analysis validated 20 of these. Significant biological functions of the differentially abundant microRNAs were apoptosis, necrosis, cell proliferation and cell invasion. Following validation, four microRNAs (miRNA‐199b‐3p, miRNA‐139‐5p, miRNA‐1839 and miRNA‐151‐5p) were detected in more than 50% of the synovial fluid samples and had higher abundance in osteoarthritic than in control joints.

**Main limitations:**

Limited sample size.

**Conclusion:**

This is the first study to determine longitudinal changes in synovial fluid microRNA abundance in an equine model of osteoarthritis. Larger studies are needed in naturally occurring osteoarthritis to interrogate putative changes identified by this study.

## 1 INTRODUCTION

MicroRNAs (miRNAs) have been the focus of extensive research over the past decade, as they have been implicated in a wide range of biological pathways. They are promising biomarker candidates for early disease detection.^1^ MiRNAs are short (18–22 nucleotides) RNA species, which are not translated into protein. Their main function is to regulate gene expression post‐transcriptionally, as miRNAs binding to their target sequence results in either translation inhibition or increased mRNA target degradation.^2^

MiRNAs are involved in the pathogenesis of osteoarthritis (OA) and could be potential OA biomarkers.^3^ OA is the most common disease affecting joints in horses and humans.^4^, ^5^ Early diagnosis of OA is challenging. Often irreversible structural changes have occurred prior to diagnosis, which is currently based primarily on clinical and radiographic manifestations. Basic molecular studies that contribute to defining and characterising OA in its earliest stages would serve to elucidate disease pathogenesis, identify novel biomarkers for early detection and identify targets for new therapies.

In horses, miRNA profiles discriminated diseased individuals from healthy in studies of rhabdomyolosis,^6^ sarcoids,^7^, ^8^, ^9^ asthma,^10^ and osteochondrosis.^11^ MiRNAs have been identified in equine cartilage,^12^ subchondral bone,^11^ and synovial fluid (SF)^13^, ^14^ including SF‐derived extracellular vesicles.^15^ In foals with osteochondrosis of the talocrural joint, five miRNAs were down‐regulated in cartilage and eight miRNAs were up‐regulated in subchondral bone, suggesting that miRNA expression might be involved in pathways important for cartilage maturation.^11^

Studies in humans^16^ and mice^17^, ^18^ suggested that a dysregulation of miRNA in OA could play a role in disease pathogenesis,^16^, ^19^ and miRNAs have been suggested to be important for cartilage function.^20^, ^21^ Moreover, miRNAs play important roles in inflammatory and pain‐related pathways in OA.^22^, ^23^, ^24^ MiRNA SF abundance differed between early and late‐stage OA in humans,^25^ and in donkeys increased abundance of miR‐146b and miR‐27b in SF was detected 1–3 months following induction of OA by intra‐articular injection of monoiodoacetate with abundance levels normalising in the later stage of the experiment (month 5–7).^26^ Nevertheless, changes in SF miRNA abundance during OA development and progression, especially in the early disease phase, have not been described.

The aim of this study was to identify miRNAs and characterise the temporal changes in their abundance in SF from horses with experimentally induced OA. We hypothesised that the abundance of miRNA would change during disease progression.

## 2 MATERIALS AND METHODS

### 2.1 Horses and study design

Nine skeletally mature Standardbred trotters (seven mares and two geldings, 2.5–7 years, 397–528 kg) were included in this study. Prior to inclusion, horses underwent clinical examination, lameness examination including flexion tests, radiographic imaging, haematological and blood‐biochemical analysis and arthrocentesis of their middle carpal joints to ensure that horses were sound and healthy.

OA was surgically induced in the left middle carpal joint, and the right middle carpal joint underwent sham surgery as described previously^4^ and detailed below. SF was sampled from the middle carpal joints before, and sequentially following OA induction.

Horses were euthanised on Day 71 or 72 with an overdose of pentobarbital sodium (140 mg/kg, Euthasol Vet, Dechra Veterinary Products). One horse, Horse 5, was euthanised according to predefined humane endpoints at Day 49, as it became lame at the walk. Following euthanasia, samples were collected from the joints.

### 2.2 Induction of osteoarthritis and exercise

Induction of OA was undertaken as described by McIlwraith^4^ with a few modifications. Under arthroscopic guidance, an osteochondral fragment was created in the left middle carpal joint by chiselling off a fragment on the dorsodistal surface of the radial carpal bone using an 8 mm curved osteotome. The fragment was left adherent to the joint capsule proximally, and an incongruent defect bed of approximately 15 mm in width was created in the exposed subchondral bone between the fragment and radial carpal bone by drilling with an arthroscopic burr. Debris from the procedure was left inside the joint. Sham surgery (arthroscopy alone) was performed in the right middle carpal joint. All portals were sutured and bandaged routinely.

From 2 weeks following OA induction and onwards, horses were exercised 5 days a week on a treadmill: 2 min trot (4.4–5.3 m/s), 2 min fast trot/gallop (9 m/s) and 2 min trot (4.4–5.3 m/s).^27^

### 2.3 Samples and analysis

SF samples were obtained from both middle carpal joints prior to surgery (Day 0) and 14, 17, 21, 28, 35, 42, 49, 56, 63 and 70 days following surgery and transferred to EDTA tubes, yielding a total of 190 samples (samples from Day 63 and 70 were missing from Horse 5, and on 4 occasions [1 OA, 3 control joins] SF could not be retrieved or sample was lost). Samples were processed within 1 h and stored at −80°C for a maximum of 6 months before small RNA sequencing and reverse transcription (RT) quantitative real‐time PCR (qPCR) analysis of miRNA abundance was performed.

### 2.4 Post‐mortem examination

Following euthanasia, both middle carpal joints were opened, and tissue samples obtained from the synovial membrane and the articular cartilage of the intermediate and third carpal bones. Tissue samples were placed in neutral buffered 10% formalin and processed routinely for haematoxylin and eosin (H&E) and safranin O (cartilage only) staining. Histological grading of the synovial membrane and cartilage was performed using the modified Mankin score as described by McIlwraith et al.^28^ In brief, synovial membrane sections were evaluated and graded for cellular infiltration, vascularity, intimal hyperplasia and subintimal oedema, where each category was graded 0–4 and the scores were added together to achieve a final histological score. Cartilage sections were graded for chondrocyte necrosis, cluster formation, fibrillation/fissuring, focal cell loss and safranin‐O stain uptake, where each category was graded 0–4 and the scores were added together to achieve a final histological score.

### 2.5 Laboratory analysis

**FIGURE 1:**
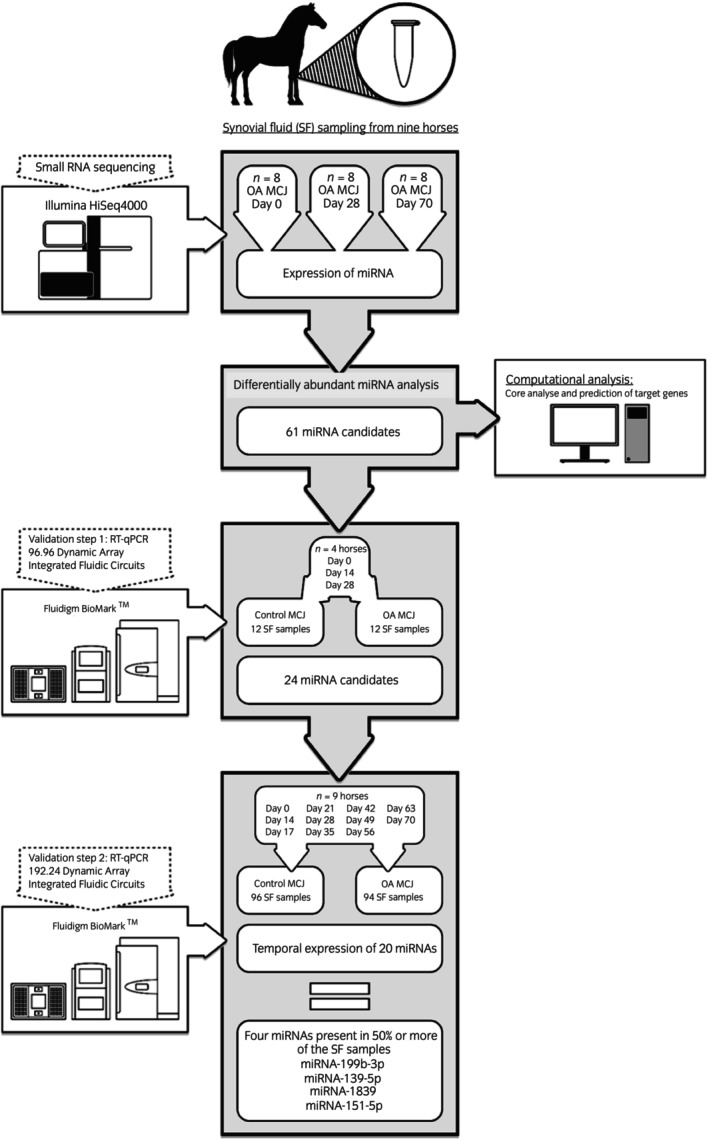
Overview of the experimental workflow. Synovial fluid (SF) samples were obtained from nine horses with experimentally induced osteoarthritis (OA) in the left middle carpal joint (MCJ) using the equine carpal osteochondral fragment model of OA.^4^ The right MCJ served as a control joint for each horse (sham surgery). SF obtained on three sampling days from eight horses underwent small RNA sequencing. MicroRNAs of interest, that is, those that were differentially abundant (DA) between Day 0, 28 and 70 (*n* = 61 miRNAs), were used for computational analysis identifying biological function and prediction of target genes. The 61 DA miRNAs were validated through reverse transcription quantitative polymerase chain reaction (RT‐qPCR), and the temporal abundance pattern mapped by assessing abundance on Day 0, 14, 17, 21, 28, 35, 42, 49, 56, 63 and 70 days after surgery. The RT‐qPCR analyses were undertaken in two steps. First, the 61 miRNAs of interest were analysed in a subset of samples from four horses, yielding 24 miRNA candidates. Second, based on the first RT‐qPCR a panel of 24 miRNAs were selected and analysed in all synovial samples obtained from all nine horses.

The laboratory workflow is depicted in Figure 1. Differentially abundant (DA) miRNAs, that is, miRNAs whose abundance at Day 28 and/or 70 differed from abundance on Day 0, were defined as miRNAs of interest. Subsequently, temporal abundance of these miRNAs was analysed using RT‐qPCR.

#### 2.5.1 RNA extraction and small RNA sequencing

SF from Horse 1 was not subjected to small RNA sequencing, as only low volumes were available, and SF obtained from this horse was retained for RT‐qPCR only.

500 μL SF was treated with a 1 μg/μL hyaluronidase (Hyaluronidase type IV, Sigma–Aldrich) as preciously described.^13^ RNA was extracted using the miRNeasy serum/plasma Advanced Kit (Qiagen) according to the manufacturer's instructions. Samples underwent on‐column DNase digestion with RNase free DNase sets (Qiagen) and RNA yield was determined by spectrophotometry (Nano Drop ND‐1000 spectrophotometer, Saveen and Werner AB).

100 ng total RNA per sample were submitted for small RNA library preparation and sequencing performed by the Centre of Genomic Research, University of Liverpool as previously described^13^ using the Illumina HiSeq4000 platform. One sample failed during laboratory processing (Horse 9 Day 0), and 23 samples thus completed small RNA sequencing.

Small RNA sequencing data was deposited in ArrayExpress (accession E‐TAB‐11840).

#### 2.5.2 Pathway analysis

Pathway analysis of DA miRNAs identified following small RNA sequencing was performed using Ingenuity Pathway Analysis (IPA) (Qiagen). First, a Core Analysis was undertaken to determine the pathways the DA miRNAs were associated with. Then potential mRNA targets of the DE miRNAs were identified using the “MicroRNA Target Filter” tool in IPA and the pathways that these target genes and their corresponding miRNAs were involved in were identified.

#### 2.5.3 Microfluidic high throughput RT‐qPCR

miRNAs profiled by qPCR were selected based on our small RNA sequencing results (Table S1) and the literature for equine and human OA. While some miRNAs were initially chosen due to their clear relevance in OA, we opted to profile all DA miRNAs with qPCR to ensure comprehensive validation. This approach allowed us to capture potentially novel or underreported miRNAs that might play critical roles in OA, to ensure that our results were robust and exploratory.

cDNA synthesis (each sample in triple technical replicates) using 10–50 ng total RNA was performed as previously described with minor modifications.^29^ cDNA was diluted 1:10 with low EDTA TE‐buffer and stored at −20°C until pre‐amplification.

Primers were designed based on the principles described by Balcells et al.,^29^ using miRprimerdesign3^30^ and synthesised by Sigma‐Aldrich (Sigma–Aldrich, Brøndby, Denmark) (Table S1).

Pre‐amplification, exonuclease treatment and qPCR were performed as previously described.^31^ Samples of cDNA were pre‐amplified using a mix of all miRNA qPCR primer pairs included in the subsequent qPCR. Pre‐amplification cycling parameters were as follows: 95°C for 10 min followed by 24 cycles of 95°C for 15 s and 60°C for 4 min. Finally, pre‐amplified exonuclease treated cDNA was diluted 1:10 with low EDTA TE‐buffer and stored at −20°C until qPCR.

MiRNAs of interest that met DA selection criteria following sequencing data analysis were tested in SF samples from four horses (Horses 2, 4, 5 and 7; Figure 1). OA and control joint samples from 3 days (Day 0, Day 14 and Day 28) were initially screened for optimal primer performance. Based on the initial screening of melt curves, amplification curves and dilution curves 24 miRNAs were selected for further qPCR analysis of their temporal abundance in SF samples from all nine horses and all time points using 192.24 dynamic arrays (Figure 1). Data were handled by the Fluidigm Real‐Time PCR Analysis software 3.0.2 (Fluidigm) and exported to GenEx5 MultiD (Västra Frölunda) for data processing as described by Brogaard et al.^31^

### 2.6 Data pre‐processing and statistical analyses

Pathology scores of OA and control joints were compared using Wilcoxon signed rank test analysed in R (R version 3.6.2 (2019‐12‐12)); *p* < 0.05 was considered significant.

Small RNA sequencing reads were aligned to the horse genome (release 90, Ensemble) using Tophat version 1.2.1.^32^ Analysis for differential miRNA abundance (comparisons between Days 0–28, 0–70, 28–70) was performed and miRNAs with *p* < 0.05, ±2‐fold change and at least five reads in at least one of the time intervals were selected for RT‐qPCR validation. Only miRNA detected in all Day 0‐samples by sequencing were included.

Log transformed values and fold changes generated following small RNA sequencing of the miRNAs of interest were used for all computational analysis, and comparisons were considered significant at *p* < 0.05.

Following qPCR and data normalisation to the mean expression of all included miRNAs data pre‐processing data were transformed from Cq into relative quantities, and the relative miRNA abundance over time was established relative to the abundance at baseline (Day 0). Samples were scaled to 1 for each individual miRNA. During pre‐processing of the qPCR results, four out of the 24 miRNAs were excluded due to poor assay efficiency calculated from three independent dilutions curves. Thus, 20 miRNAs were evaluated for DA over time.

The presence of miRNAs expression in OA and control samples was analysed, taking into account the correlation between joints on the same horse as well as correlation over time. Relative quantities were log transformed in order to enable a normal distribution for further statistical analysis. Changes in the temporal abundance levels of miRNAs with detectable abundance in >50% of SF samples were analysed in SAS Enterprise Guide 7.1 (SAS Institute Inc.) using a mixed model, with time as a factor (i.e., no linearity over time assumed), and the two legs were allowed to have different time paths (interaction term leg * time) and only restricted to be equal at baseline (before OA induction). The correlation structure considered the serial correlation over time for each leg (autoregressive of first order), as well as the correlation between legs of the same animal. Finally model fit was determined by graphical assessment of distribution of residuals. In case of a nonsatisfactory model fit (residuals not normally distributed), a nonparametric method for paired data (Wilcoxon signed rank test) was performed to compare miRNA abundance levels at selected timepoints.

## 3 RESULTS

### 3.1 Histology

**FIGURE 2:**
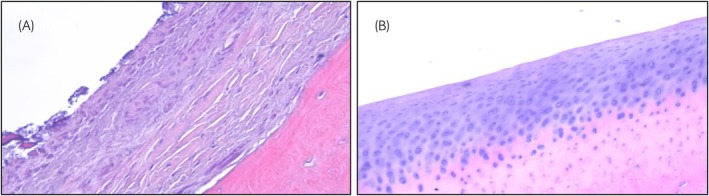
Histological images experimental induction of osteoarthritis (OA). Representative images from third carpal bone (haematoxylin and eosin staining) 70 days after surgical induction of OA. (A) Articular cartilage from OA joint and (B) articular cartilage from control joint. In the OA joint complete loss of articular cartilage is present, and fibrillating fibrocartilage has replaced the hyaline cartilage.

Synovial membrane scores at euthanasia (Table 1) showed significantly greater cellular infiltration, intimal hyperplasia and subintimal oedema in the OA joints compared with control joints. The final score of the synovial membrane was significantly higher (*p* < 0.05) in the OA joints (Table 1). Histological evaluation of the cartilage from the third carpal bone (Table 1) showed significantly greater chondrocyte necrosis, cluster formation and focal cell loss scores in OA joints than in control joints. Vascularity and subintimal fibrosis did not differ. Furthermore, the final histological score of the third carpal bone was significantly higher in OA joints than in control joints (*p* < 0.05) (Figure 2, Table 1).

**TABLE 1:** Histological scores (modified Mankin score scores as described by Mcilwraith et al.^28^) at 70 days after experimental induction of osteoarthritis (OA) in one middle carpal joint in nine horses.

		OA joint	Control joint	
Histologic examination	Outcome	Modified Mankin score (median [range])	Modified Mankin score (median [range])	*p*‐value
Synovial membrane	Cellular infiltration	3 (1–4)	1 (1–3)	0.03
	Vascularity	3 (1–4)	2 (1–3)	0.2
	Intimal hyperplasia	3 (1–4)	1 (1–2)	0.04
	Subintimal oedema	3 (0–4)	1 (0–2)	0.01
	Subintimal fibrosis	4 (2–4)	3 (2–4)	0.3
	Final pathology score synovial membrane	15 (4–19)	8 (6–12)	0.02
Cartilage	Chondrocyte necrosis Third carpal bone	4 (1–4)	0 (0–1)	0.03
	Chondrocyte necrosis Intermediate carpal bone	4 (0–4)	0 (0–4)	0.1
	Cluster formation Third carpal bone	2 (2–4)	0 (0–0)	0.03
	Cluster formation Intermediate carpal bone	4 (1–4)	1 (0–4)	0.1
	Fibrillation/fissuring Third carpal bone	1 (0–3)	0 (0–2)	0.3
	Fibrillation/fissuring Intermediate carpal bone	3 (1–4)	1 (0–4)	>0.9
	Focal cell loss Third carpal bone	4 (1–4)	0 (0–1)	0.03
	Focal cell loss Intermediate carpal bone	4 (0–4)	1 (0–4)	0.3
	Safranin O stain uptake Third carpal bone	3 (2–4)	1 (0–4)	0.06
	SO stain uptake Intermediate carpal bone	4 (3–4)	4 (0–4)	0.2
	Final pathology score Third carpal bone	15 (7–18)	1.5 (0–5)	0.04
	Final pathology score Intermediate carpal bone	17 (5–20)	7 (2–16)	0.06

*Note*: Control joint was the contralateral middle carpal joint, which underwent sham surgery (arthroscopy alone).

### 3.2 Small RNA sequencing and computational analysis

Small RNA sequencing identified 295 miRNAs in SF (Table S2). The subsequent DA analysis revealed 61 miRNAs (Table S1), all of these were DA on Day 28 compared with Day 0. On Day 70, none of the identified miRNAs were DA compared with Day 0 or 28.

**FIGURE 3:**
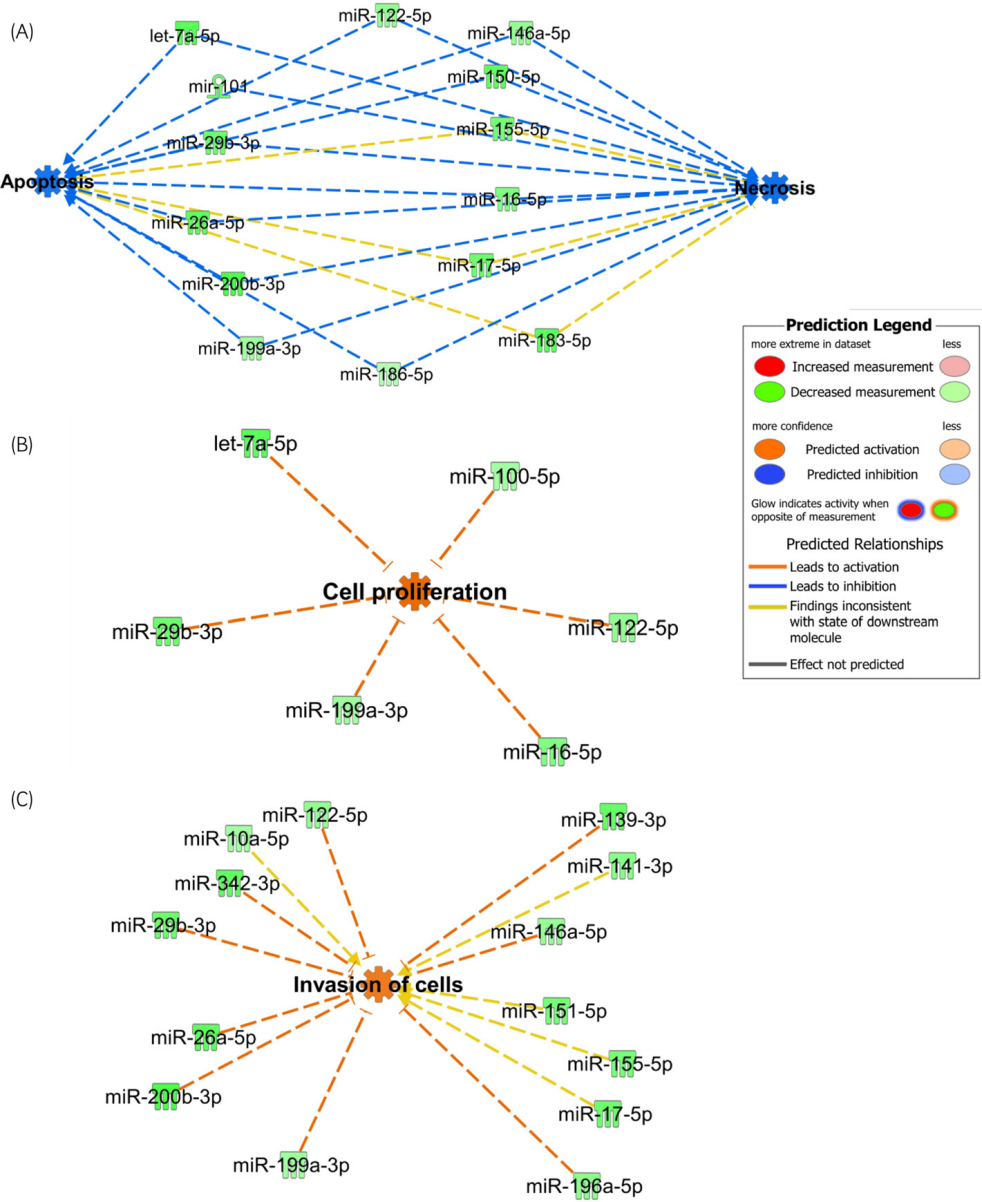
Core analysis of 61 differentially abundant miRNAs identified in synovial fluid from horses with experimentally induced osteoarthritis. The most substantial biological functions identified were (A) apoptosis and necrosis, both of which were predicted to be decreased, (B) cell proliferation and (C) cell invasion, with the latter two predicted to be increased. Key to the main features in the networks is shown. Image made in Ingenuity Pathway Analysis.

IPA of the 61 DA miRNAs were input into a Core Analysis. The most substantial biological functions with significant activation *z*‐scores were apoptosis (*p* = 1.31E−6; *z* = −1.6, Figure 3A) and necrosis (*p* = 0.006; *z* = −1.8, Figure 3A), both predicted to be inhibited; cell proliferation (*p* = 1.5E−11; *z* = 2.1, Figure 3B) and cell invasion (*p* = 3.8E−8; *z* = 1.5, Figure 3C), both predicted to be increased. Additionally, 16 miRNAs were associated with fibrosis (*p* = 1.4E−13).

Using the “MicroRNA Target Filter” tool in IPA we identified mRNA targets for 39 of the 61 miRNAs. 404 putative protein coding genes were predicted to be regulated by these miRNAs. We then input this network of miRNAs and their mRNA targets back into IPA for a Core Analysis. The main upstream regulators for these mRNAs were predicted to be tumour necrosis factor (*TNF*) (*p* = 1.17E−113), beta‐oestradiol (*p* = 1.17E−108), transforming growth factor beta 1 (*TGFB1*) (*p* = 2.75E−108) and interleukin 1 beta (*IL1B*) (*p* = 5.23E−92).

### 3.3 Microfluidic high‐throughput RT‐qPCR

Microfluidic qPCR was able to identify 20 out of 61 DA miRNAs: let‐7c, let 7d‐5p, let 7e, let 7f, miRNA‐23a, miRNA‐27b, miRNA‐30d, miRNA‐98, miRNA‐101, miRNA‐125a‐3p, miRNA‐139‐5p, miRNA‐148b‐5p, miRNA‐151‐5p, miRNA‐155‐5p, miRNA‐196b‐5p, miRNA‐199b‐3p, miRNA‐200a, miRNA‐409‐3p, miRNA‐499‐5p and miRNA‐1839.

Twenty of these miRNAs were detectable in 4%–71% of the SF samples (Table 2). In total, 190 SF samples obtained from nine horses at 11 time points 0–70 days after experimental induction of OA (96 samples from OA joints, 94 samples from sham operated control joints) were included in the table.

**TABLE 2:** MicroRNAs (miRNAs) detectable in synovial fluid samples obtained from nine horses with experimentally induced osteoarthritis (OA).

MicroRNA	Overall number (%) of samples with detectable abundance	Number (%) of OA samples with detectable abundance	Number (%) of control samples with detectable abundance	*p*‐value (difference between OA and control)
miRNA‐148b‐5p	59 (31.05)	26 (27.08)	33 (35.11)	0.3
miRNA‐499‐5p	8 (4.21)	8 (8.51)	0 (0.00)	–
miRNA‐199b‐3p	**135 (71.05)**	**75 (78.13)**	**60 (63.83)**	**0.07**
miRNA‐155‐5p	15 (7.89)	7 (7.29)	8 (8.51)	0.8
miRNA‐151‐5p	**104 (54.74)**	**58 (60.42)**	**46 (48.95)**	**0.2**
let‐7d‐5p	93 (48.95)	56 (58.33)	37 (39.36)	0.08
let‐7e	93 (48.95)	54 (56.25)	39 (41.49)	0.2
let‐7c	89 (46.84)	51 (53.13)	38 (40.43)	0.3
miRNA‐1839	**108 (56.84)**	**50 (52.08)**	**58 (61.70)**	**0.33**
let‐7f	78 (41.05)	48 (50.00)	30 (31,91)	0.1
miRNA‐139‐5p	**124 (65.26)**	**47 (48.96)**	**77 (81.91)**	**0.002**
miRNA‐23a	64 (33.68)	46 (47.92)	18 (19.15)	0.06
miRNA‐30d	68 (35.79)	42 (43.75)	26 (27.66)	0.05
miRNA‐98	51 (26.84)	34 (35.42)	17 (18.09)	0.03
miRNA‐125a‐3p	65 (34.21)	23 (23.96)	42 (44.68)	0.02
miRNA‐200a	28 (14.74)	19 (19.79)	9 (9.57)	0.2
miRNA‐27b	54 (28.42)	19 (19.79)	35 (37.23)	0.1
miRNA‐196b‐5p	53 (27.89)	18 (18.75)	35 (37.23)	0.1
miRNA‐101	29 (15.26)	16 (16.67)	13 (13.83)	0.6
miRNA‐409‐3p	45 (23.68)	15 (15.63)	30 (31.91)	0.1

*Note*: MicroRNAs with detectable abundance in more than 50% of all samples are highlighted in bold.

**FIGURE 4:**
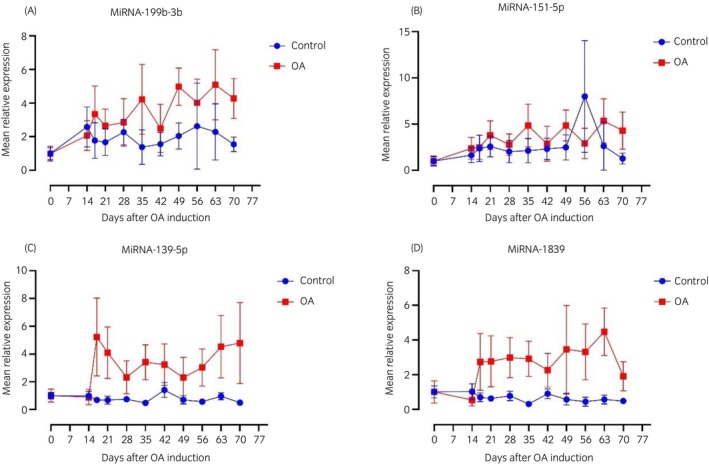
Temporal abundance of four microRNAs (miRNA). These were present in >50% of synovial fluid samples obtained from nine horses with experimentally induced osteoarthritis (OA), (A) miRNA‐199b‐3b, (B) miRNA‐151‐5p, (C) miRNA‐139‐5p and (D) miRNA‐1839. Sham operated (arthroscopy alone) joints served as controls. Mean relative abundance ±SEM (error bars are depicted). Image made in Prism (GraphPad).

MiRNA‐199b‐3p, miRNA‐139‐5p, miRNA‐151‐5p and miRNA‐1839 were detectable in more than 50% (54.7%–71.1%) of samples (Table 2), and the temporal changes were analysed further (Figure 4). A statistical mixed model analysis revealed that miRNA‐199b‐3p was significantly higher in OA joints (Figure 4A), however the model fit was not completely satisfactory, and significance was lost following a confirmatory nonparametric test. MiRNA‐98 and miRNA‐30d were detected more frequently in OA samples, whereas miRNA‐139‐5p and miRNA‐125‐3p were detected significantly more frequently in the control samples (Table 2). MiRNA‐98, miRNA‐30d and miRNA‐125‐3p were detected in less than 50% of the samples, but differing significantly between OA and control joints. The temporal patterns of miRNA‐98, miRNA‐30d and miRNA‐125‐3p are shown in Figure S1.

## 4 DISCUSSION

This study was the first to identify and map temporal miRNA abundance patterns in equine SF during the development of OA. Histological evaluation revealed significant cartilage degradation and synovial membrane inflammation in OA joints, confirming the development of OA in our model.^4^ Small RNA sequencing demonstrated 61 DA miRNAs in equine SF at Day 28 compared with Day 0. In contrast, no miRNAs were DA in late‐stage OA (Day 70), similar to previous findings in humans^33^ and donkeys,^26^ where miRNAs were found to be expressed in the early stages of OA. This early change in miRNA abundance (Day 28) was partly confirmed using RT‐qPCR. We showed sustained alterations in miRNA abundance from Day 14 and onwards, thus suggesting that these changes were associated with OA development and not with the arthroscopic procedure alone. The equine carpal chip model specifically models the post‐traumatic phenotype of OA, and as such, every event from carpal chipping to erosion of joint cartilage is considered part of the disease pathogenesis.

The abundance of 20 miRNAs was detected by RT‐qPCR, and three miRNAs (miRNA‐199b‐3b, miRNA‐139‐5p and miRNA‐1839) were consistently more abundant in SF isolated from OA joints than in control joints (Figure 4A,C,D). There is no consensus regarding miRNA up‐ and down‐regulation patterns in joint disease. Previous studies in equidae and humans identified a variety of miRNAs isolated from plasma,^34^ serum,^26^, ^35^ SF^13^, ^14^, ^16^, ^25^, ^26^ and articular tissues from OA‐affected individuals.^36^, ^37^ The most consistently identified miRNAs in previous studies were miRNA‐146a, miRNA‐155, and let‐7 family,^35^, ^37^, ^38^, ^39^, ^40^ and these miRNAs may be associated with increased apoptosis, increased production of pro‐inflammatory cytokines and pain modulation in OA.^22^, ^41^, ^42^, ^43^ These miRNAs were also DA in SF in our small RNA sequencing data. Further, by using qPCR we were able to assess abundance of let‐7c let‐7d, let‐7e and let‐7f in 41.1%–49.0% of the SF samples. Abundance of miRNA‐155 was found in less than 10% of the SF samples, whereas miRNA‐146a was below the limits of detection using qPCR. The difference between our findings and those of previous studies and the inability to detect these latter two miRNAs in our study could be explained by several factors. These include differences in sample material, species, sex, disease duration, severity and stage and different phenotypes of OA. This underpins the complexity of identifying and validating miRNA biomarker candidates. Other studies have failed to identify significant changes in miRNA in OA. Serum miRNAs were not associated with cartilage damage in early post‐traumatic OA in a mouse model^17^ or in human patients with anterior cruciate ligament injury.^44^ Interestingly, we recently identified an upregulation of miRNA‐146a expression (as well as expression of miRNA‐150 and miRNA‐409‐3p) in articular cartilage from horses with experimental OA,^37^ and the relationship between tissue and secreted miRNA species thus warrants further exploration.

In our study miRNA‐199b‐3b was detectable in more than 70% of SF samples and elevated in OA SF (Figure 4A). MiRNA‐199 was identified following sequencing by Ali et al.,^33^ where miRNA‐199a‐3b was one out of 11 known and novel miRNAs that were DA in plasma from patients with early OA. MiRNA‐199 is present in chondrocytes, and regulates cyclooxygenase 2 expression^45^ and chondrocyte ageing.^46^

Another of the four most abundant miRNAs in this study was miRNA‐139‐5p. Interestingly, this miRNA was detected in a significantly higher number of control joints samples, but levels were higher in SF from OA joints, corresponding to results from a recent study on experimentally induced OA in horses, where expression levels were higher in articular cartilage with a high pathological score.^37^ The significance of a dose–response relationship between miRNA and pathophysiological events remains to be elucidated, and it is thus currently unclear how the amount of miRNA present affects tissue responses. A previous study suggested that miRNA‐139 may be involved in OA pathogenesis, as it was up‐regulated in human OA cartilage.^47^ MiR‐139 is involved in pathophysiological processes related to OA, as it inhibited chondrocyte proliferation and migration^47^ and induced apoptosis in chondrocytes from humans with OA.^48^

Chondrocyte death and thereby degradation of the extracellular matrix cause destruction of the articular cartilage,^49^ and cell death and apoptosis are increased in OA cartilage.^50^ Interestingly, pathway analysis of the 61 DA miRNAs identified in our study suggested decreased apoptosis and necrosis in the early phase of OA development. This was surprising, as previous research demonstrated an association between cartilage degradation and increased chondrocyte apoptosis and necrosis.^50^, ^51^ MiRNAs including miR‐146a can target and downregulate pro‐apoptotic genes, thus reducing apoptosis in joint tissues. This miRNA is often up‐regulated in OA and can inhibit inflammatory responses by targeting signalling molecules such as TNF Receptor‐Associated Factor 6 and 1 Interleukin‐1 Receptor‐Associated Kinase 1, involved in the NF‐κB pathway. This modulation may lead to reduced apoptosis in chondrocytes and synovial cells. Furthermore, miRNAs may influence necrosis‐related pathways by affecting genes involved in cellular stress responses or autophagy; for example miR‐155 in our data. MiR‐155 targets proteins involved in the regulation of necroptosis potentially reducing necrotic cell death in OA. Some miRNAs have anti‐inflammatory roles, which could indirectly decrease cell death. For instance, miRNAs that suppress the expression of pro‐inflammatory cytokines may reduce inflammation‐driven cell death pathways, both apoptotic and necrotic. We hypothesise that in OA, the expression profiles of altered SF miRNAs reflects an adaptive response to joint stress. This altered expression could result in the down‐regulation of pro‐apoptotic or pro‐necrotic miRNAs, promoting cell survival in a degraded joint environment.

Together with the increased cell infiltration and proliferation, our pathway analyses indicated that the SF‐derived miRNAs could be associated with repair mechanisms or counteract cell and tissue degradation in the very early phase of OA. Moreover, predicted target genes were involved in the inflammatory processes, including TNFα, TGFβ1 and IL‐1β important in OA pathogenesis.^52^ The pro‐inflammatory cytokines TNFα and IL‐1β are key drivers of inflammatory processes and initiators of cartilage degradation.^52^, ^53^ Increased concentrations of TNFα and IL‐1β were found in SF from horses with traumatic OA^54^ and in articular tissues from horses with naturally occurring carpal OA.^55^ Thus, miRNAs may be important factors in fine tuning cytokine expression during OA in order to ensure the robust regulation of pro‐inflammatory gene expression.

This study had several limitations. First, profiling of miRNA in cell‐free biological fluids is challenging due to the low abundance of miRNA. This had a significant impact on the results of the present study, in which miRNA abundance failed to reach the limits of detection in several of the samples. These are known as ‘missing values’ which complicate statistical analyses through their influence on model fit and the assumption of normal distribution. In addition, it was difficult to know if the values missing were due to a biological effect (truly not there) or due to depth of sequencing. Indeed, confirming results with nonparametric analyses resulted in loss of significance. Second, as we were interested in temporal changes, the power was low in terms of the number of horses used in the study. We did not perform a sample size calculation. Sample size affects reproducibility in miRNA biomarkers studies when using sensitive high‐throughput screening techniques. A small sample size increases the risk of false negative results and true biomarker candidates may be missed.^56^ Third, the platform used can influence the results.^57^ Using small RNA sequencing, we were able to detect miRNA present in equine SF without prior knowledge of transcript information. Small RNA sequencing reproducibility can vary due to the complexity of this platform.^57^ Validation of small RNA sequencing data using RT‐qPCR increases the robustness of the results. This would ideally be undertaken in an independent cohort, challenging in large animal studies. A fourth, nontechnical limitation of the study pertains to the animal model used. OA is a heterogenic disease with distinct phenotypes. In humans, several OA phenotypes have been described, including inflammatory, hormonal, genetic, post‐traumatic and metabolic phenotypes.^58^ While the equine carpal osteochondral fragment model does not encompass all pathophysiological aspects of these phenotypes, the model offers a controlled setup with known time of onset and a singular cause of OA, which facilitates studies aimed at studying early temporal disease progression. A great advantage of the model is that the size of the equine middle carpal joint permits repeated sampling over time, thus limiting use of experimental animals and permitting assessment of temporal changes. Finally we did not correct the *p*‐values for multiple comparisons in the assessment of DA miRNAs between the three timepoints in the RNAseq analysis. This was because we used this as an interim screening step to decide which miRNAs to include in our final, confirmatory, quantitative RT‐qPCR analysis that served as a validation of the RNAseq results.

Further mechanistic studies of the miRNAs identified in this study are required to uncover the roles of miRNA‐199b‐3p, miRNA‐139‐5p, miRNA‐151‐5p and miRNA‐1839 in the pathogenesis of OA and validate their biological function and biomarker potential.

## 5 CONCLUSION

This is the first equine study mapping temporal abundance patterns of miRNAs in SF during OA initiation and progression. The abundance of miRNA‐199b‐3p, miRNA‐139‐5p, miRNA‐151‐5p and miRNA‐1839 were detectable in the majority of the SF samples, and their abundance in OA joints was elevated from Day 14 and throughout the study period. Computational analyses revealed potential biological pathways and target genes that could be relevant to OA pathogenesis in horses.

## FUNDING INFORMATION

This work was made possible through support from the Independent Research Fund Denmark, Technology and Production Sciences (grant number DFF‐7017‐00066, 2017), the Horse Levy Foundation, and the Gerda and Aage Haensch's Foundation. Furthermore, Lægefonden—AP Møller Foundation, Kuustos Foundation and Toosbuys Foundation provided financial support for the animal experiment. MW is funded by a PhD scholarship jointly awarded by the University of Copenhagen, the Technical University of Denmark and the Swedish University of Agricultural Science. MJP was funded through a Wellcome Trust Clinical Intermediate Fellowship (grant 107 471/Z/15/Z) and supported by Versus Arthritis as part of the MRC Versus Arthritis Centre for Integrated Musculoskeletal Ageing.

## CONFLICT OF INTEREST STATEMENT

The authors declare no conflicts of interest.

## AUTHOR CONTRIBUTIONS

**Marie Walters:** Conceptualization; methodology; data curation; writing – original draft; writing – review and editing; funding acquisition; formal analysis; validation; visualization. **Kerstin Skovgaard:** Writing – review and editing; methodology; data curation. **Peter M. H. Heegaard:** Conceptualization; methodology; writing – review and editing. **Yongxiang Fang:** Formal analysis; data curation; software; methodology. **Yalda A. Kharaz:** Writing – review and editing; formal analysis; data curation. **Louise Bundgaard:** Conceptualization; data curation; methodology; writing – review and editing. **Lene T. Skovgaard:** Formal analysis; writing – review and editing. **Henrik E. Jensen:** Writing – review and editing; methodology. **Pia H. Andersen:** Conceptualization; writing – review and editing; methodology; data curation. **Mandy J. Peffers:** Writing – review and editing; data curation; formal analysis; funding acquisition; methodology; visualization. **Stine Jacobsen:** Supervision; conceptualization; investigation; funding acquisition; formal analysis; data curation; writing – review and editing; resources; project administration; visualization.

## DATA INTEGRITY STATEMENT

Stine Jacobson had full access to all the data in the study and takes responsibility for the integrity of the data and the accuracy of data analysis.

## ETHICAL ANIMAL RESEARCH

The experimental protocol was approved by the Danish Animal Experiments Inspectorate (permit 2017‐15‐0201‐01314) and by the local ethical committee of the Large Animal Teaching Hospital of University of Copenhagen. The procedures were carried out according to EU Directive 2010/63/EU for animal experiments and in accordance with the Danish Animal Testing Act.

## INFORMED CONSENT

Not applicable.

### PEER REVIEW

The peer review history for this article is available at https://www.webofscience.com/api/gateway/wos/peer‐review/10.1111/evj.14456.

## Supporting information


**Figure S1.** Temporal expression pattern of the three microRNAs (miRNAs).

**Table S1.** Primer list of the 61 miRNAs of interest.

**Table S2.** Table of all miRNAs identified in synovial fluid.

## Data Availability

The data that support the findings of this study are openly available in ArrayExpress at https://www.ebi.ac.uk/biostudies/arrayexpress/studies/E-MTAB-11840?query=e-mtab-11840, reference number E‐MTAB‐11840.

## References

[1] De Guire V , Robitaille R , Tetreault N , Guerin R , Menard C , Bambace N , et al. Circulating miRNAs as sensitive and specific biomarkers for the diagnosis and monitoring of human diseases: promises and challenges. Clin Biochem. 2013;46(10–11):846–860. 23562576 10.1016/j.clinbiochem.2013.03.015

[2] O'Brien J , Hayder H , Zayed Y , Peng C . Overview of microRNA biogenesis, mechanisms of actions, and circulation. Front Endocrinol (Lausanne). 2018;9:402. 30123182 10.3389/fendo.2018.00402 PMC6085463

[3] Miyaki S , Asahara H . Macro view of microRNA function in osteoarthritis. Nat Rev Rheumatol. 2012;8(9):543–552. 22890245 10.1038/nrrheum.2012.128 PMC3572197

[4] McIlwraith CW , Frisbie DD , Kawcak CE . The horse as a model of naturally occurring osteoarthritis. Bone Joint Res. 2012;1(11):297–309. 23610661 10.1302/2046-3758.111.2000132 PMC3626203

[5] Loeser RF , Goldring SR , Scanzello CR , Goldring MB . Osteoarthritis: a disease of the joint as an organ. Arthritis Rheum. 2012;64(6):1697–1707. 22392533 10.1002/art.34453 PMC3366018

[6] Barrey E , Bonnamy B , Barrey EJ , Mata X , Chaffaux S , Guerin G . Muscular microRNA expressions in healthy and myopathic horses suffering from polysaccharide storage myopathy or recurrent exertional rhabdomyolysis. Equine Vet J. 2010;42(S38):303–310. 10.1111/j.2042-3306.2010.00267.x 21059022

[7] Unger L , Abril C , Gerber V , Jagannathan V , Koch C , Hamza E . Diagnostic potential of three serum microRNAs as biomarkers for equine sarcoid disease in horses and donkeys. J Vet Intern Med. 2021;35(1):610–619. 33415768 10.1111/jvim.16027 PMC7848377

[8] Unger L , Gerber V , Pacholewska A , Leeb T , Jagannathan V . MicroRNA fingerprints in serum and whole blood of sarcoid‐affected horses as potential non‐invasive diagnostic biomarkers. Vet Comp Oncol. 2019;17(1):107–117. 30430738 10.1111/vco.12451

[9] Unger L , Jagannathan V , Pacholewska A , Leeb T , Gerber V . Differences in miRNA differential expression in whole blood between horses with sarcoid regression and progression. J Vet Intern Med. 2019;33(1):241–250. 30506726 10.1111/jvim.15375 PMC6335546

[10] Hulliger MF , Pacholewska A , Vargas A , Lavoie JP , Leeb T , Gerber V , et al. An integrative miRNA–mRNA analysis reveals striking transcriptomic similarities between severe equine asthma and specific asthma Endotypes in humans. Genes (Basel). 2020;11(10):1143. 32998415 10.3390/genes11101143 PMC7600650

[11] Desjardin C , Vaiman A , Mata X , Legendre R , Laubier J , Kennedy SP , et al. Next‐generation sequencing identifies equine cartilage and subchondral bone miRNAs and suggests their involvement in osteochondrosis physiopathology. BMC Genomics. 2014;15(1):798. 25227120 10.1186/1471-2164-15-798 PMC4190437

[12] Peffers M , Liu X , Clegg P . Transcriptomic signatures in cartilage ageing. Arthritis Res Ther. 2013;15(4):R98. 23971731 10.1186/ar4278 PMC3978620

[13] Castanheira C , Balaskas P , Falls C , Ashraf‐Kharaz Y , Clegg P , Burke K , et al. Equine synovial fluid small non‐coding RNA signatures in early osteoarthritis. BMC Vet Res. 2021;17(1):26. 33422071 10.1186/s12917-020-02707-7 PMC7796526

[14] Baker ME , Lee S , Clinton M , Hackl M , Castanheira C , Peffers MJ , et al. Investigation of microRNA biomarkers in equine distal interphalangeal joint osteoarthritis. Int J Mol Sci. 2022;23(24):15526. 36555166 10.3390/ijms232415526 PMC9779011

[15] Anderson JR , Jacobsen S , Walters M , Bundgaard L , Diendorfer A , Hackl M , et al. Small non‐coding RNA landscape of extracellular vesicles from a post‐traumatic model of equine osteoarthritis. Front Vet Sci. 2022;9:901269. 36003409 10.3389/fvets.2022.901269 PMC9393553

[16] Xu JF , Zhang SJ , Zhao C , Qiu BS , Gu HF , Hong JF , et al. Altered microRNA expression profile in synovial fluid from patients with knee osteoarthritis with treatment of hyaluronic acid. Mol Diagn Ther. 2015;19(5):299–308. 26232909 10.1007/s40291-015-0155-2

[17] Kung LH , Zaki S , Ravi V , Rowley L , Smith MM , Bell KM , et al. Utility of circulating serum miRNAs as biomarkers of early cartilage degeneration in animal models of post‐traumatic osteoarthritis and inflammatory arthritis. Osteoarthr Cartil. 2017;25(3):426–434. 10.1016/j.joca.2016.09.002 27621213

[18] Castanheira C , Anderson JR , Fang Y , Milner PI , Goljanek‐Whysall K , House L , et al. Mouse microRNA signatures in joint ageing and post‐traumatic osteoarthritis. Osteoarthr Cartil Open. 2021;3(4):100186. 34977596 10.1016/j.ocarto.2021.100186 PMC8683752

[19] Ali SA , Peffers MJ , Ormseth MJ , Jurisica I , Kapoor M . The non‐coding RNA interactome in joint health and disease. Nat Rev Rheumatol. 2021;17(11):692–705. 34588660 10.1038/s41584-021-00687-y

[20] Kobayashi T , Lu J , Cobb BS , Rodda SJ , McMahon AP , Schipani E , et al. Dicer‐dependent pathways regulate chondrocyte proliferation and differentiation. Proc Natl Acad Sci U S A. 2008;105(6):1949–1954. 18238902 10.1073/pnas.0707900105 PMC2538863

[21] Ntoumou E , Tzetis M , Braoudaki M , Lambrou G , Poulou M , Malizos K , et al. Serum microRNA array analysis identifies miR‐140‐3p, miR‐33b‐3p and miR‐671‐3p as potential osteoarthritis biomarkers involved in metabolic processes. Clin Epigenetics. 2017;9:127. 29255496 10.1186/s13148-017-0428-1 PMC5728069

[22] Li X , Gibson G , Kim JS , Kroin J , Xu S , van Wijnen AJ , et al. MicroRNA‐146a is linked to pain‐related pathophysiology of osteoarthritis. Gene. 2011;480(1–2):34–41. 21397669 10.1016/j.gene.2011.03.003 PMC3095758

[23] Chang ZK , Meng FG , Zhang ZQ , Mao GP , Huang ZY , Liao WM , et al. MicroRNA‐193b‐3p regulates matrix metalloproteinase 19 expression in interleukin‐1beta‐induced human chondrocytes. J Cell Biochem. 2018;119(6):4775–4782. 29323744 10.1002/jcb.26669

[24] Yamasaki K , Nakasa T , Miyaki S , Ishikawa M , Deie M , Adachi N , et al. Expression of MicroRNA‐146a in osteoarthritis cartilage. Arthritis Rheum. 2009;60(4):1035–1041. 19333945 10.1002/art.24404 PMC2670476

[25] Li YH , Tavallaee G , Tokar T , Nakamura A , Sundararajan K , Weston A , et al. Identification of synovial fluid microRNA signature in knee osteoarthritis: differentiating early‐ and late‐stage knee osteoarthritis. Osteoarthr Cartil. 2016;24(9):1577–1586. 10.1016/j.joca.2016.04.019 27143365

[26] Yassin AM , AbuBakr HO , Abdelgalil AI , Farid OA , El‐Behairy AM , Gouda EM . Circulating miR‐146b and miR‐27b are efficient biomarkers for early diagnosis of Equidae osteoarthritis. Sci Rep. 2023;13(1):7966. 37198318 10.1038/s41598-023-35207-3 PMC10192321

[27] Frisbie DD , Kawcak CE , McIlwraith CW . Evaluation of the effect of extracorporeal shock wave treatment on experimentally induced osteoarthritis in middle carpal joints of horses. Am J Vet Res. 2009;70(4):449–454. 19335099 10.2460/ajvr.70.4.449

[28] McIlwraith CW , Frisbie DD , Kawcak CE , Fuller CJ , Hurtig M , Cruz A . The OARSI histopathology initiative—recommendations for histological assessments of osteoarthritis in the horse. Osteoarthr Cartil. 2010;18(Suppl 3):S93–S105. 10.1016/j.joca.2010.05.031 20864027

[29] Balcells I , Cirera S , Busk PK . Specific and sensitive quantitative RT‐PCR of miRNAs with DNA primers. BMC Biotechnol. 2011;11(1):70. 21702990 10.1186/1472-6750-11-70 PMC3135530

[30] Busk PK . A tool for design of primers for microRNA‐specific quantitative RT‐qPCR. BMC Bioinform. 2014;15(1):29. 10.1186/1471-2105-15-29 PMC3922658 24472427

[31] Brogaard L , Heegaard PM , Larsen LE , Mortensen S , Schlegel M , Dürrwald R , et al. Late regulation of immune genes and microRNAs in circulating leukocytes in a pig model of influenza A (H1N2) infection. Sci Rep. 2016;6:21812. 26893019 10.1038/srep21812 PMC4759598

[32] Kim D , Salzberg SL . TopHat‐fusion: an algorithm for discovery of novel fusion transcripts. Genome Biol. 2011;12(8):R72. 21835007 10.1186/gb-2011-12-8-r72 PMC3245612

[33] Ali SA , Gandhi R , Potla P , Keshavarzi S , Espin‐Garcia O , Shestopaloff K , et al. Sequencing identifies a distinct signature of circulating microRNAs in early radiographic knee osteoarthritis. Osteoarthr Cartil. 2020;28(11):1471–1481. 10.1016/j.joca.2020.07.003 32738291

[34] Borgonio Cuadra VM , Gonzalez‐Huerta NC , Romero‐Cordoba S , Hidalgo‐Miranda A , Miranda‐Duarte A . Altered expression of circulating microRNA in plasma of patients with primary osteoarthritis and in silico analysis of their pathways. PLoS One. 2014;9(6):e97690. 24901787 10.1371/journal.pone.0097690 PMC4046959

[35] Beyer C , Zampetaki A , Lin NY , Kleyer A , Perricone C , Iagnocco A , et al. Signature of circulating microRNAs in osteoarthritis. Ann Rheum Dis. 2015;74(3):e18. 24515954 10.1136/annrheumdis-2013-204698

[36] Diaz‐Prado S , Cicione C , Muinos‐Lopez E , Hermida‐Gomez T , Oreiro N , Fernandez‐Lopez C , et al. Characterization of microRNA expression profiles in normal and osteoarthritic human chondrocytes. BMC Musculoskelet Disord. 2012;13:144. 22883423 10.1186/1471-2474-13-144 PMC3495209

[37] Andersen C , Walters M , Bundgaard L , Berg LC , Vonk LA , Lundgren‐Åkerlund E , et al. Intraarticular treatment with integrin α10β1‐selected mesenchymal stem cells affects microRNA expression in experimental post‐traumatic osteoarthritis in horses. Front Vet Sci. 2024;11:1374681.F. 38596460 10.3389/fvets.2024.1374681 PMC11002141

[38] Murata K , Furu M , Yoshitomi H , Ishikawa M , Shibuya H , Hashimoto M , et al. Comprehensive microRNA analysis identifies miR‐24 and miR‐125a‐5p as plasma biomarkers for rheumatoid arthritis. PLoS One. 2013;8(7):e69118. 23874885 10.1371/journal.pone.0069118 PMC3715465

[39] Murata K , Yoshitomi H , Tanida S , Ishikawa M , Nishitani K , Ito H , et al. Plasma and synovial fluid microRNAs as potential biomarkers of rheumatoid arthritis and osteoarthritis. Arthritis Res Ther. 2010;12(3):R86. 20470394 10.1186/ar3013 PMC2911870

[40] Churov AV , Oleinik EK , Knip M . MicroRNAs in rheumatoid arthritis: altered expression and diagnostic potential. Autoimmun Rev. 2015;14(11):1029–1037. 26164649 10.1016/j.autrev.2015.07.005

[41] Feng L , Feng C , Wang CX , Xu DY , Chen JJ , Huang JF , et al. Circulating microRNA let‐7e is decreased in knee osteoarthritis, accompanied by elevated apoptosis and reduced autophagy. Int J Mol Med. 2020;45(5):1464–1476. 32323821 10.3892/ijmm.2020.4534 PMC7138275

[42] Kurowska‐Stolarska M , Alivernini S , Ballantine LE , Asquith DL , Millar NL , Gilchrist DS , et al. MicroRNA‐155 as a proinflammatory regulator in clinical and experimental arthritis. Proc Natl Acad Sci U S A. 2011;108(27):11193–11198. 21690378 10.1073/pnas.1019536108 PMC3131377

[43] Li X , Kroin JS , Kc R , Gibson G , Chen D , Corbett GT , et al. Altered spinal microRNA‐146a and the microRNA‐183 cluster contribute to osteoarthritic pain in knee joints. J Bone Miner Res. 2013;28(12):2512–2522. 23744481 10.1002/jbmr.2002 PMC4361038

[44] Zhang L , Yang M , Marks P , White LM , Hurtig M , Mi QS , et al. Serum non‐coding RNAs as biomarkers for osteoarthritis progression after ACL injury. Osteoarthr Cartil. 2012;20(12):1631–1637. 10.1016/j.joca.2012.08.016 PMC3478481 22944527

[45] Akhtar N , Haqqi TM . MicroRNA‐199a* regulates the expression of cyclooxygenase‐2 in human chondrocytes. Ann Rheum Dis. 2012;71(6):1073–1080. 22294637 10.1136/annrheumdis-2011-200519 PMC4509731

[46] Ukai T , Sato M , Akutsu H , Umezawa A , Mochida J . MicroRNA‐199a‐3p, microRNA‐193b, and microRNA‐320c are correlated to aging and regulate human cartilage metabolism. J Orthop Res. 2012;30(12):1915–1922. 22674437 10.1002/jor.22157

[47] Hu W , Zhang W , Li F , Guo F , Chen A . miR‐139 is up‐regulated in osteoarthritis and inhibits chondrocyte proliferation and migration possibly via suppressing EIF4G2 and IGF1R. Biochem Biophys Res Commun. 2016;474(2):296–302. 27105918 10.1016/j.bbrc.2016.03.164

[48] Makki MS , Haqqi TM . miR‐139 modulates MCPIP1/IL‐6 expression and induces apoptosis in human OA chondrocytes. Exp Mol Med. 2015;47(10):e189. 26450708 10.1038/emm.2015.66 PMC4673474

[49] Hwang HS , Kim HA . Chondrocyte apoptosis in the pathogenesis of osteoarthritis. Int J Mol Sci. 2015;16(11):26035–26054. 26528972 10.3390/ijms161125943 PMC4661802

[50] Sharif M , Whitehouse A , Sharman P , Perry M , Adams M . Increased apoptosis in human osteoarthritic cartilage corresponds to reduced cell density and expression of caspase‐3. Arthritis Rheum. 2004;50(2):507–515. 14872493 10.1002/art.20020

[51] Zamli Z , Sharif M . Chondrocyte apoptosis: a cause or consequence of osteoarthritis? Int J Rheum Dis. 2011;14(2):159–166. 21518315 10.1111/j.1756-185X.2011.01618.x

[52] Sellam J , Berenbaum F . The role of synovitis in pathophysiology and clinical symptoms of osteoarthritis. Nat Rev Rheumatol. 2010;6(11):625–635. 20924410 10.1038/nrrheum.2010.159

[53] Berenbaum F . The quest for the Holy Grail: a disease‐modifying osteoarthritis drug. Arthritis Res Ther. 2007;9(6):111. 18096086 10.1186/ar2335 PMC2246245

[54] Bertuglia A , Pagliara E , Grego E , Ricci A , Brkljaca‐Bottegaro N . Pro‐inflammatory cytokines and structural biomarkers are effective to categorize osteoarthritis phenotype and progression in Standardbred racehorses over five years of racing career. BMC Vet Res. 2016;12(1):246. 27821120 10.1186/s12917-016-0873-7 PMC5100096

[55] Kamm JL , Nixon AJ , Witte TH . Cytokine and catabolic enzyme expression in synovium, synovial fluid and articular cartilage of naturally osteoarthritic equine carpi. Equine Vet J. 2010;42(8):693–699. 21039798 10.1111/j.2042-3306.2010.00140.x PMC4183755

[56] Kok MGM , de Ronde MWJ , Moerland PD , Ruijter JM , Creemers EE , Pinto‐Sietsma SJ . Small sample sizes in high‐throughput miRNA screens: a common pitfall for the identification of miRNA biomarkers. Biomol Detect Quantif. 2018;15:1–5. 29276692 10.1016/j.bdq.2017.11.002 PMC5737945

[57] Pritchard CC , Cheng HH , Tewari M . MicroRNA profiling: approaches and considerations. Nat Rev Genet. 2012;13(5):358–369. 22510765 10.1038/nrg3198 PMC4517822

[58] Van Spil WE , Kubassova O , Boesen M , Bay‐Jensen AC , Mobasheri A . Osteoarthritis phenotypes and novel therapeutic targets. Biochem Pharmacol. 2019;165:41–48. 30831073 10.1016/j.bcp.2019.02.037

