# Supplementary material for: Identification and characterisation of temporal abundance of microRNAs in synovial fluid from an experimental equine model of osteoarthritis
Source: Equine Vet J. 2025 Jan 8;57(4):1138–50. doi: 10.1111/evj.14456 (PMC12135755; doi:10.1111/evj.14456)
Supplement: Supplementary file 1 — Figure S1. Temporal expression pattern of the three microRNAs (miRNAs). [file EVJ-57-1138-s003.pdf]

Figure S1: Temporal expression pattern of the three microRNAs (miRNAs), which were detectable in less than 50 % of synovial fluid (SF) samples, but were detected significantly more frequently in either the osteoarthritic (OA) joint (miRNA-30d and miRNA-98) or the control joint (miRNA-125a). SF samples were obtained repeatedly during the 70-day study period from nine horses with experimentally induced OA in one middle carpal joint. The contralateral joint served as control (sham surgery). Expression was assessed using quantitative reverse transcription polymerase chain reaction (RT-qPCR). Mean of the relative expression from nine horses ±standard error of the mean are depicted.

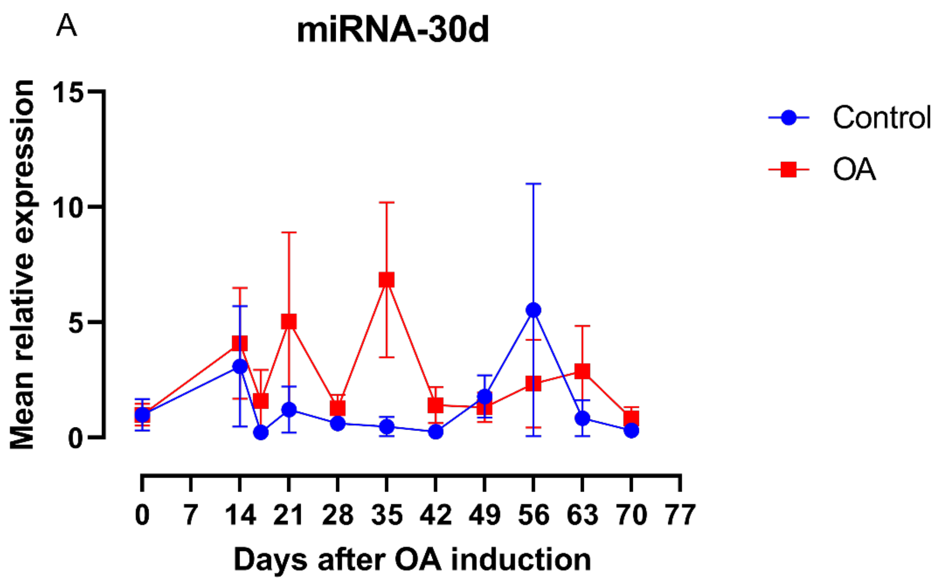

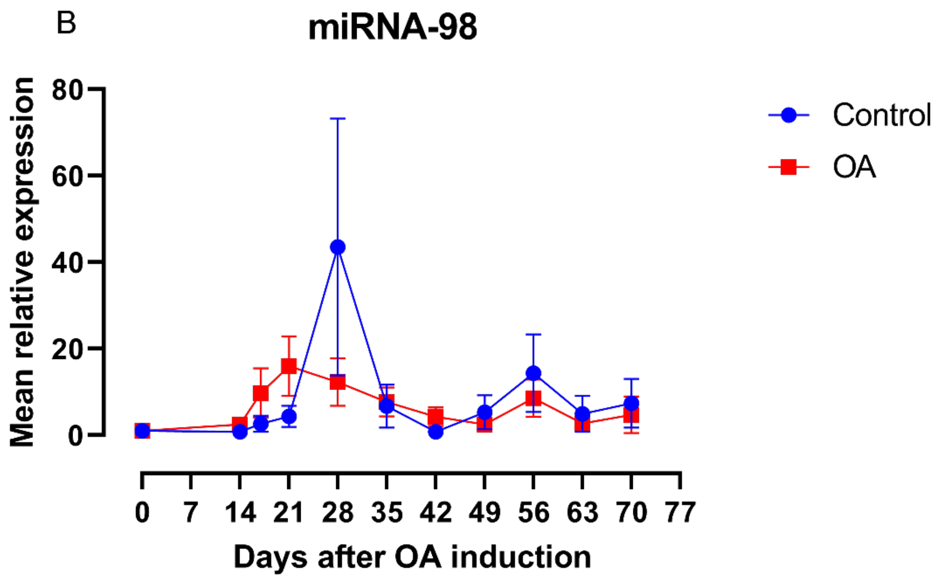
B
miRNA-98
Mean relative expression
80
60
40
20
0
0 7 14 21 28 35 42 49 56 63 70 77
Days after OA induction
Control
OA

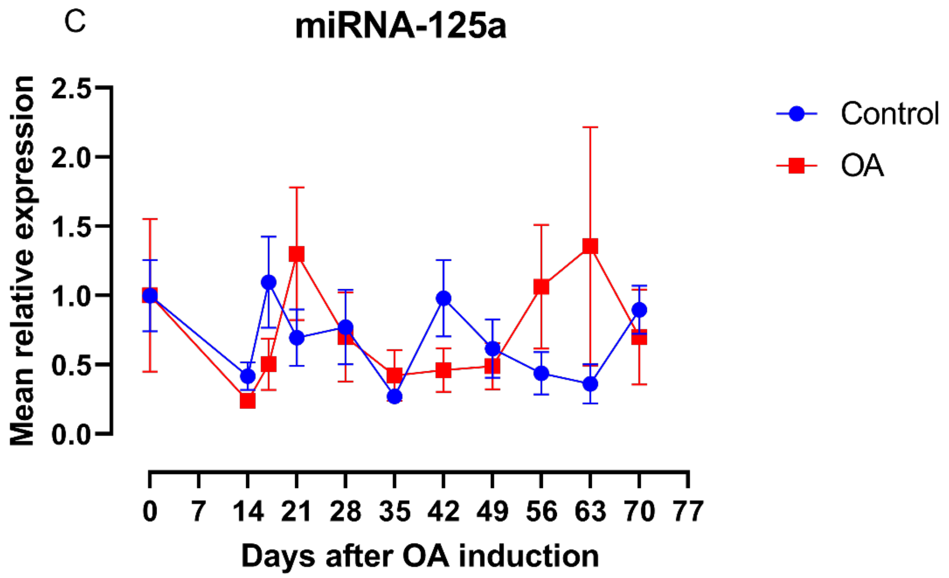
C
miRNA-125a
Mean relative expression
2.5
2.0
1.5
1.0
0.5
0.0
0 7 14 21 28 35 42 49 56 63 70 77
Days after OA induction
Control
OA
