# Supplementary material for: Identification and characterisation of temporal abundance of microRNAs in synovial fluid from an experimental equine model of osteoarthritis
Source: Equine Vet J. 2025 Jan 8;57(4):1138–50. doi: 10.1111/evj.14456 (PMC12135755; doi:10.1111/evj.14456)
Supplement: Supplementary file 2 — Table S1. Primer list of the 61 miRNAs of interest. [file EVJ-57-1138-s001.pdf]

Table S1. Primer list of the 61 miRNAs of interest.

Gene_id	Gene_name	Sequence_smallRNAseq	Primer sequence (Sequence 5' to 3')
**GeneID:100314799**	eca-miR-183	TATGGCACTGGTAGAATTCACT	F_GCAGTATGGCACTGGTAGA<br>R_TCCAGTTTTTTTTTTTTTTTCAGTGA
**GeneID:100314986<br>/GeneID:100314805**	eca-miR-101	TACAGTACTGTGATAACTGAA	F_GCGCAGTACAGTACTGTGA<br>R_GGTCCAGTTTTTTTTTTTTTTTCAGTT
**GeneID:100314971**	eca-miR-139	5p TCTACAGTGCACGTGTCTCCAG<br>3p GGAGACGCGGCCCTGTTGGAGT	F_CTACAGTGCACGTGTCTC<br>R_CCAGTTTTTTTTTTTTTTCTGGAG<br>F_CTACAGTGCACGTGTCTC<br>R_GGTCCAGTTTTTTTTTTTTTTCTG
**GeneID:100315003**	eca-miR-182	TTTGGCAATGGTAGAACTCACACTG	F_AGTTTGGCAATGGTAGAACTC<br>R_CCAGTTTTTTTTTTTTTTCAGTGTG<br>F_TTGGCAATGGTAGAACTCAC<br>R_GGTCCAGTTTTTTTTTTTTTTTCAG
**GeneID:100315074**	eca-let-7f	TGAGGTAGTAGATTGTATAGTT	F_CGCAGTGAGGTAGTAGATTG<br>R_GGTCCAGTTTTTTTTTTTTTTTAACTATAC
**GeneID:100314994**	eca-miR-155	TTAATGCTAATCGTGATAGGGGT	F_CGCAGTTAATGCTAATTGTGATAG<br>R_CCAGTTTTTTTTTTTTTTCCCCTA
**GeneID:100314942**	eca-miR-374a	TTATAATACAACCTGATAAGTG	F_AGCGCAGTTATAATACAACCTG<br>R_GGTCCAGTTTTTTTTTTTTTTCACT

**GeneID:100314892**	eca-miR-27b	TTCACAGTGGCTAAGTTCTGC	F_CAGTTCACAGTGGCTAAGTTC<br>R_TCCAGTTTTTTTTTTTTTTTGCAGA
**GeneID:100314974**	eca-let-7e	TGAGGTAGGAGGTTGTATAGTT	F_CAGTGAGGTAGGAGGTTGT<br>R_GGTCCAGTTTTTTTTTTTTTTTAACTATAC
**GeneID:100314909**	eca-miR-409	5p AGGTTACCCGAGCAACTTTGCAT<br>3p GAATGTTGCTCGGTGAACCCCT	F_GGAATGTTGCTCGGTGAAC<br>R_AGGTCCAGTTTTTTTTTTTTTTTAGG<br>F_GGTTACCCGAGCAACTTTG<br>R_GGTCCAGTTTTTTTTTTTTTTTATGC
**GeneID:100314870**<br>**/GeneID:100314818**	eca-miR-26a	TTCAAGTAATCCAGGATAGGCT	F_CGAGTTCAAGTAATCCAGGA<br>R_CCAGTTTTTTTTTTTTTTTAGCCTATC
**GeneID:100314874**	eca-miR-19a	TGTGCAAATCTATGCAAAACTGA	F_GCAGTGTGCAAATCTATGC<br>R_GGTCCAGTTTTTTTTTTTTTTTCAG
**GeneID:100314889**	eca-let-7d	GAGGTAGTAGGTTGCATAGTT	F_CAGAGAGGTAGTAGGTTGCAT<br>R_GGTCCAGTTTTTTTTTTTTTTTAACTATG<br>F_GCAGAGAGGTAGTAGGTTGC<br>R_GGTCCAGTTTTTTTTTTTTTTTAACTATG
**GeneID:100314867**	eca-let-7g	TGAGGTAGTAGTTTGTACAGTT	F_CGCAGTGAGGTAGTAGTTTG<br>R_GTCCAGTTTTTTTTTTTTTTTAACTGT
**GeneID:100314800**	eca-miR-29a	TAGCACCATCTGAAATCGGTTA	F_GCTAGCACCATCTGAAATCG

			R_TCCAGTTTTTTTTTTTTTTTAACCGA
**GeneID:100315035**	eca-miR-1839	AAGGTAGATAGAACAGGTCTTG	F_GCAGAAGGTAGATAGAACAGGTC<br>R_GTCCAGTTTTTTTTTTTTTTTCAAGAC
**GeneID:100314902**	eca-miR-342	5p: AGGGGTGCTATCTGTGATTGAG<br>3p:TCTCACACAGAAATCGCACCCGT	F_CAGAGGGGTGCTATCTGT<br>R_TCCAGTTTTTTTTTTTTTTTCAATCAC<br>F_GTCTCACACAGAAATCGCA<br>R_CCAGTTTTTTTTTTTTTTTACGGGT
**GeneID:100315100**	eca-miR-196b	TAGGTAGTTTCCTGTTGTTGGG	F_GCAGTTCACAGTGGCTAAG<br>R_CAGTTTTTTTTTTTTTTTGCGGAA<br>F_CAGTTCACAGTGGCTAAGTTC<br>R_CAGTTTTTTTTTTTTTTTGCGGAA
**GeneID:100314816**	eca-miR-148b	5p: GAAGTTCTGTTATACACTCAGG<br>3p: TCAGTGCATCACAGAACTTTGT	F_GCAGGAAGTTCTGTTATACACTC<br>R_CCAGTTTTTTTTTTTTTTTCCTGAG<br>F_CGCAGGAAGTTCTGTTATACAC<br>R_CAGTTTTTTTTTTTTTTTCCTGAGTG<br>F_AGTCAGTGCATCACAGAAC<br>R_GGTCCAGTTTTTTTTTTTTTTTACAAAG
**GeneID:100315038**	eca-miR-200b	TAATACTGCCTGGTAATGATGA	F_CGCAGTAATACTGCCTGGT<br>R_GGTCCAGTTTTTTTTTTTTTTTCATCA<br>F_CGCAGTAATACTGCCTGGT

			R_GGTCCAGTTTTTTTTTTTTTTTCATC
**GeneID:104797028**	eca-miR-329b	AACGAACCTGGTTAACCTCTTTT	F_CGCAGAACGAACCTGGT<br>R_GGTCCAGTTTTTTTTTTTTTTTAAAAGAG<br>F_CGCAGAACGAACCTGGT<br>R_GTCCAGTTTTTTTTTTTTTTTAAAAGAGG
**GeneID:100314824**	eca-miR-27a	TTCACAGTGGCTAAGTTCCGC	F_CAGTTCACAGTGGCTAAGA<br>R_CAGTTTTTTTTTTTTTTTGCGGAA
**GeneID:100315102**	eca-miR-122	TGGAGTGTGACAATGGTGTTTG	F_GTGGAGTGTGACAATGGTG<br>R_GTCCAGTTTTTTTTTTTTTTTACAAACA
**GeneID:100314833**	eca-miR-151	TCGAGGAGCTCACAGTCTAGT	F_TCGAGGAGCTCACAGTC<br>R_GTCCAGTTTTTTTTTTTTTTTACTAGAC<br>F_TCGAGGAGCTCACAGTC<br>R_GGTCCAGTTTTTTTTTTTTTTTACTAGA
**GeneID:100314852**	eca-miR-192	CTGACCTATGAATTGACAGCC	F_GCAGCTGACCTATGAATTGAC<br>R_CAGTTTTTTTTTTTTTTTGGCTGTC
**GeneID:100315026**	eca-let-7c	TGAGGTAGTAGGTTGTATGGTT	F_ GCAGTGAGGTAGTAGGTTGTA<br>R_GTCCAGTTTTTTTTTTTTTTTAACCA
**GeneID:100314926**	eca-miR-99a	AACCCGTAGATCCGATCTTGTG	F_CAGAACCCGTAGATCCGA<br>R_TCCAGTTTTTTTTTTTTTTTCACAAGA
**GeneID:100314875**	eca-miR-20a	TAAAGTGCTTATAGTGCAGGTAG	F_CGCAGTAAAGTGCTTATAGTG

			R_GTCCAGTTTTTTTTTTTTTTTTACCTG
**GeneID:100314861**	eca-miR-146a	TGAGAACTGAATTCCATGGGTT	F_GCAGTGAGAACTGAATTCCA<br>R_GGTCCAGTTTTTTTTTTTTTTTTAACC
**GeneID:100314835**	eca-miR-125a	5p:TCCCTGAGACCCTTTAACCTGTGA<br>3p: ACAGGTGAGGTTCTTGGGAGCC	F_CCCTGAGACCCTTTAACCT<br>R_GGTCCAGTTTTTTTTTTTTTTTTCAC<br>F_CCCTGAGACCCTTTAACCT<br>R_GTCCAGTTTTTTTTTTTTTTTTCACAG<br>F_CAGGTGAGGTTCTTGGGA<br>R_TCCAGTTTTTTTTTTTTTTTTGGCT<br>F_GACAGGTGAGGTTCTTGG<br>R_GTTTTTTTTTTTTTTTTGGCTCCCA
**GeneID:104795234**	eca-miR-676	CCGTCCTAAGGTTGTTGAGTT	F_GCCGTCCTAAGGTTGTTGA<br>R_GGTCCAGTTTTTTTTTTTTTTTTAACTC<br>F_GCCGTCCTAAGGTTGTTGA<br>R_GGTCCAGTTTTTTTTTTTTTTTTAACTCA
**GeneID:100314895**	eca-miR-872	AAGGTTACTTGTTAGTTCAGG	F_CGCAGAAGGTTACTTGTTAGTTC<br>R_GGTCCAGTTTTTTTTTTTTTTTTCCT<br>F_CGCAGAAGGTTACTTGTTAGTTC<br>R_GTCCAGTTTTTTTTTTTTTTTTCCTGA
**GeneID:100314776**	eca-miR-184	TGGACGGAGAACTGATAAGGGT	F_CAGTGGACGGAGAACTGA

			R_GTCCAGTTTTTTTTTTTTTTTACCCT
**GeneID:100315031**	eca-miR-98	TGAGGTAGTAAGTTGTATTGTT	F_ CGCAGTGAGGTAGTAAGTTGT<br>R_AGGTCCAGTTTTTTTTTTTTTTTAACA
**GeneID:100315010**	eca-miR-150	TCTCCCAACCCTTGTACCAGTG	F_GTCTCCCAACCCTTGTAC<br>R_GTCCAGTTTTTTTTTTTTTTTCACTG
**GeneID:100315047**	eca-miR-29b	TAGCACCATTTGAAATCAGTGTT	F_CAGTAGCACCATTTGAAATCAGT<br>R_GGTCCAGTTTTTTTTTTTTTTTAACACT
**GeneID:100315055**	eca-miR-30b	TGTAAACATCCTACACTCAGCT	F_GCAGTGTAAACATCCTACACTC<br>R_CCAGTTTTTTTTTTTTTTTAGCTGAG
**GeneID:100315027**<br>**/GeneID:100314820**	eca-let-7a	TGAGGTAGTAGGTTGTATAGTT	F_GCAGTGAGGTAGTAGGTTGT<br>R_GTCCAGTTTTTTTTTTTTTTTAACT
**GeneID:104795156**	eca-miR-1307	ACTCGGCGTGGCGTCGGTCGTGGTA	F_GCGTGGCGTCGGT<br>R_TCCAGTTTTTTTTTTTTTTTCACGA
**GeneID:100315065**	eca-miR-340	5p: TTATAAAGCAATGAGACTGATT<br>3p: TCCGTCTCAGTTACTTTATAGCC	F_CGCAGTTATAAAGCAATGAGAC<br>R_GGTCCAGTTTTTTTTTTTTTTTAATCAG<br>F_CGCAGTTATAAAGCAATGAGAC<br>R_GTCCAGTTTTTTTTTTTTTTTAATCAGTC<br>F_GCAGTCCGTCTCAGTTAC<br>R_TCCAGTTTTTTTTTTTTTTTGGCT<br>F_GCAGTCCGTCTCAGTTAC

			R_GTCCAGTTTTTTTTTTTTTTTGGCTA
**GeneID:100314797**	eca-miR-148a	TCAGTGCACTACAGAACTTTGT	F_CAGTCAGTGCACTACAGAAC<br>R_GGTCCAGTTTTTTTTTTTTTTTTACAAAG<br>F_GCAGTCAGTGCACTACAG<br>R_GGTCCAGTTTTTTTTTTTTTTTTACAAAG
**GeneID:100314983**	eca-miR-10b	TACCCTGTAGAACCGAATTTGTG	F_CAGTACCCTGTAGAACCGA<br>R_GGTCCAGTTTTTTTTTTTTTTTCAC<br>F_CAGTACCCTGTAGAACCGA<br>R_GGTCCAGTTTTTTTTTTTTTTTCACA
**GeneID:100314888**	eca-miR-499	5p: TTAAGACTTGCAGTGATGTTT<br>3p: AACATCACAGCAAGTCTGTGCT	F_GCAGTTAAGACTTGCAGTGA<br>R_GGTCCAGTTTTTTTTTTTTTTTAAACATC<br>F_GCAGTTAAGACTTGCAGTGA<br>R_GTCCAGTTTTTTTTTTTTTTTAAACATCA<br>F_AGAACATCACAGCAAGTCTG<br>R_GGTCCAGTTTTTTTTTTTTTTTAGCA<br>F_AGAACATCACAGCAAGTCTG<br>R_CCAGTTTTTTTTTTTTTTTAGCACAG
**GeneID:100314931**	eca-miR-215	ATGACCTATGAATTGACAGAC	F_CGCAGATGACCTATGAATTGAC<br>R_GGTCCAGTTTTTTTTTTTTTTTGTC

**GeneID:100314984 /GeneID:100315039**	eca-miR-30c	TGTAAACATCCTACACTCTCAGC	F_CAGTGTAAACATCCTACACTCTC<br>R_CCAGTTTTTTTTTTTTTTTGCTGAG
**GeneID:100314887**	eca-miR-296	GAGGGTTGGGTGGAGGCTTTCC	F_GGCCCCCCCCAATC<br>R_GTCCAGTTTTTTTTTTTTTTTACAGGA
**GeneID:100314922**	eca-miR-199b	5p: CCCAGTGTTTAGACTATCTGTTC 3p: ACAGTAGTCTGCACATTGGTTA	F_CCCAGTGTTCAGACTACCT<br>R_GTCCAGTTTTTTTTTTTTTTTGAACAG<br>F_CCCAGTGTTCAGACTACCT<br>R_CCAGTTTTTTTTTTTTTTTGAACAGG
**GeneID:100315006**	eca-miR-200c	TAATACTGCCGGGTAATGATGGA	F_AGTAATACTGCCGGGTAATGA<br>R_GGTCCAGTTTTTTTTTTTTTTTCCA<br>F_AGTAATACTGCCGGGTAATGA<br>R_TCCAGTTTTTTTTTTTTTTTCCATCA
**GeneID:100315002**	eca-miR-140	5p: CAGTGGTTTTACCCTATGGTAG 3p: TACCACAGGGTAGAACCACGG	F_CAGAGTGGTTTTACCCTATGG<br>R_GTCCAGTTTTTTTTTTTTTTTCTACC
**GeneID:100314784**	eca-miR-200a	TAACACTGTCTGGTAACGATGT	F_CAGTAACACTGTCTGGTAACG<br>R_CCAGTTTTTTTTTTTTTTTAACATCGT
**GeneID:100314808**	eca-miR-186	CAAAGAATTCTCCTTTTGGGCT	F_CAGCAAAGAATTCTCCTTTTGG<br>R_CCAGTTTTTTTTTTTTTTTAAGCCCA

**GeneID:100314878**	eca-miR-28	5p: AAGGAGCTCACAGTCTATTGAG 3p: CACTAGATTGTGAGCTCCTGGA	F_CAGAAGGAGCTCACAGTCT<br>R_GGTCCAGTTTTTTTTTTTTTTTCTC<br><br>F_CAGAAGGAGCTCACAGTCT<br>R_GGTCCAGTTTTTTTTTTTTTTTCTCA<br><br>F_CGCAGCACTAGATTGTGAG<br>R_GGTCCAGTTTTTTTTTTTTTTTCCA<br><br>F_CGCAGCACTAGATTGTGAG<br><br>R_CCAGTTTTTTTTTTTTTTTCCAGGA
**GeneID:100315058**	eca-miR-195	TAGCAGCACAGAAATATTGGC	F_CGCAGTAGCAGCACAGA<br>R_TCCAGTTTTTTTTTTTTTTTGCCA
**GeneID:100315057**	eca-miR-10a	TACCCTGTAGATCCGAATTTGTG	F_GCAGTACCCTGTAGATCCGA<br>R_GGTCCAGTTTTTTTTTTTTTTTACAAATTC
**GeneID:104795096**	eca-miR-1388	AGGACTGTCCAACCTGAGAATGGT	F_AGGACTGTCCAACCTGAG<br>R_GGTCCAGTTTTTTTTTTTTTTTACCA
**GeneID:100314823**	eca-miR-23a	ATCACATTGCCAGGGATTTCC	F_CATCACATTGCCAGGGAT<br>R_CGTCCAGTTTTTTTTTTTTTTTGGAA
**GeneID:104797037**	eca-miR-8992	CGCGCTGCAGGCTGCGCCCGGGGA	F_CGCGCTGCAGGCT<br>R_GGTCCAGTTTTTTTTTTTTTTTCCC

**GeneID:100314834**	eca-miR-30d	TGTAAACATCCCCGACTGGAAG	F_GTAAACATCCCCGACTGGA<br>R_CCAGTTTTTTTTTTTTTTTAGCTTCC
**GeneID:104795223**	eca-miR-7177b	AAATGGTCCCCTAGTGCTCTGG	F_CAGTAAATGATCCCCTGGTG<br>R_GGTCCAGTTTTTTTTTTTTTTTCTAGG<br>F_GAAATGGTCCCCTAGTGCT<br>R_GGTCCAGTTTTTTTTTTTTTTTCCA
**GeneID:100314811**	eca-miR-214	ACAGCAGGCACAGACAGGCAGT	F_CAGCAGGCACAGACAG<br>R_CAGGTCCAGTTTTTTTTTTTTTTTACT<br>F_ACAGCAGGCACAGACA<br>R_CAGGTCCAGTTTTTTTTTTTTTTTACT
**GeneID:100314863**	eca-miR-378	ACTGGACTTGGAGTCAGAAGG	F_AGACTGGACTTGGAGTCAG<br>R_CAGTTTTTTTTTTTTTTTGCCTTCTG
